# Concentration of Fe(3+)-Triapine in BEAS-2B Cells

**DOI:** 10.3390/ijms20123062

**Published:** 2019-06-22

**Authors:** William E. Antholine, Charles R. Myers

**Affiliations:** 1 Department of Biophysics, Medical College of Wisconsin, Milwaukee, WI 53226, USA; 2 Department of Pharmacology and Toxicology, Medical College of Wisconsin, Milwaukee, WI 53226, USA; cmyers@mcw.edu

**Keywords:** electron paramagnetic resonance, EPR, Triapine, BEAS-2B bronchial epithelial cells

## Abstract

An electron paramagnetic resonance (EPR) method was used to determine the concentration of the antitumor agent Triapine in BEAS-2B cells when Triapine was bound to iron (Fe). Knowledge of the concentration of Fe-Triapine in tumor cells may be useful to adjust the administration of the drug or to adjust iron uptake in tumor cells. An EPR spectrum is obtained for Fe(3+)-Triapine, Fe(3+)(Tp)_2_^+^, in BEAS-2B cells after addition of Fe(3+)(Tp)_2_^+^. Detection of the low spin signal for Fe(3+)(Tp)_2_^+^ shows that the Fe(3+)(Tp)_2_^+^ complex is intact in these cells. It is proposed that Triapine acquires iron from transferrin in cells including tumor cells. Here, it is shown that iron from purified Fe-transferrin is transferred to Triapine after the addition of ascorbate. To our knowledge, this is the first time that the EPR method has been used to determine the concentration of an iron antitumor agent in cells.

## 1. Introduction

**Figure 1:**
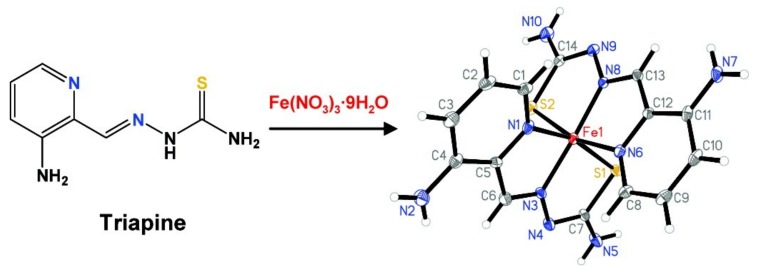
Schematic of FeTp_2_ designated Fe-Triapine. Reprinted with permission from Kowol, C.R.; Trondl, R.; Heffeter, P.; Arion, V.B.; Jukupec, M.A.; Roller, A., Galanski, M.; Berger, W.; Keppler, B.K. Impact of metal coordination on cytotoxicity of 3-aminopyridine-2-carboxaldehyde thiosemicarbazone (Triapine) and novel insights into terminal dimethylation, *J. Med. Chem.*, 2009, 52, 5032–5043, doi: 10.1021/jm900528d). Copyright 2009 American Chemical Society.

The treatment of locally advanced cervical cancer has been successful with a combination of radiation therapy, a weekly dose of cisplatin, and a three-times weekly dose of Triapine (Tp) [1,2]. However, we are not aware of other clear-cut successes from the 19 Phase 1 and 2 trials using Triapine. The efficacy of Triapine, an antitumor agent, may improve the formation and location of Fe(3+)-Triapine by varying the iron in a cell, for example, by adding an iron supplement, by sequestration of iron primarily from Fe-Transferrin (FeTf), by increasing iron absorption using a hemochromatosis cell line, or by displacing iron from FeTf by the addition of gallium [3,4,5,6]. A schematic for Fe(3+)-Triapine, Fe(3+)(Tp)_2_^+^, is shown in Figure 1.

The electron paramagnetic resonance (EPR) spectrum of the 2-formyl pyridine monothiosemicarbazonato cupric complex indicates that it forms adducts between complex and amino acids from the cells [7,8]. There is a slow destruction of the cupric complex with a first order decay of 4.5 × 10^5^ when 0.1 mM of the cupric complex is incubated with 1–15 mg of cell protein per milliliter [7]. We expected Fe(2+)-Triapine to be reduced until all the reducing equivalents were depleted or until oxygen was depleted. However, Fe(3+)-Triapine exists under our conditions and under the conditions for uptake in SW480 cells [9]. We have found no evidence for FeTpX where X is covalently bound. The log of the formation constant of a highly stable bis-ligated iron-Triapine complex is logβ[Fe(3+)(Tp)2]+ equals 26.6 [9,10], which is similar to the log formation constant for the iron complex using the parent ligand [7]. K2 > K1 indicates that Fe(3+)(Tp)_2_^+^ and Fe(2+)(Tp)_2_ are the major complexes. Therefore, we would expect to find Fe(3+)(Tp)_2_^+^ and Fe(2+)(Tp)_2_ in biological systems. In a previous study, we showed that Triapine (6–12 µM for 72 h) is very effective at causing mitochondrial redox stress at this low dose [11]. The uptake of Fe-Triapine using the EPR method has already been reported for SW480 cells [9]. In this communication, we discuss the EPR method to quantitate Fe(3+)-Triapine in cells. Most likely, this method is applicable to isolated mitochondria.

Moreover, because lung tumors often do not respond to conventional treatment over the long term, approaches to improve the effectiveness of Triapine would be welcome and would include coordinating the administration of Triapine and manipulating the influx and efflux of iron. Previously, we treated A549 cells, which are found in lung tissue, with Triapine [11]. Treatment of A549 cells with Triapine may indirectly account for the oxidation of peroxiredoxin-3 in these treated cells [11]. Here, an EPR method was used to measure the concentration of Fe(3+)(Tp)_2_^+^ in BEAS-2B cells and to demonstrate the transfer of Fe from FeTf to Triapine to form Fe(3+)(Tp)_2_^+^. This EPR method measures the concentration of Fe(3+)(Tp)_2_^+^ and could be valuable for future studies that try to manipulate the formation of Fe-Triapine in cells. 

## 2. Results and Discussion

### 2.1. Using an EPR Method to Determine the Concentration of Fe(3+)(Tp)_2_^+^ in BEAS-2B Cells 

**Figure 2:**
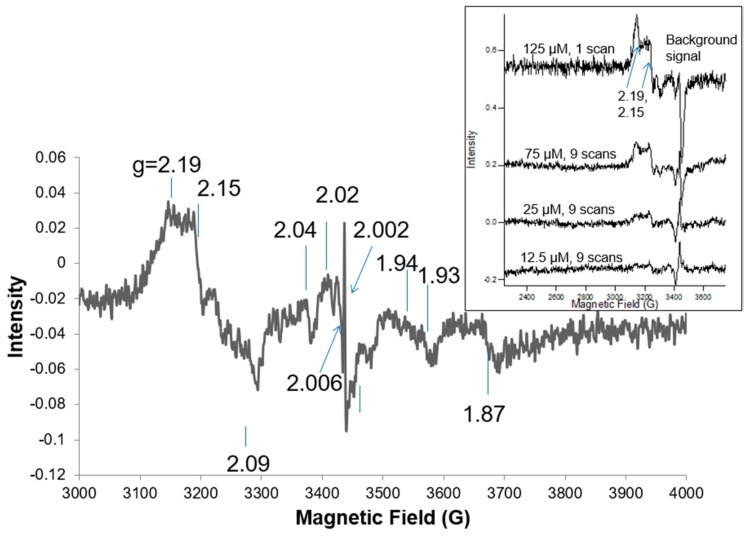
The electron paramagnetic resonance (EPR) spectra of BEAS-2B cells (6 × 10^7^ cells/mL) treated with Fe(3+)(Tp)_2_^+^ (33 µM concentration in cells). Insert: The EPR signal for Fe(3+)(Tp)_2_^+^ at different concentrations. Spectrometer conditions, 5 G mod.; microwave freq., 9.633 GHz; 7 K; 25 scans; microwave power, 0.2 mW.

Following the addition of Fe(3+)(Tp)_2_^+^ to BEAS-2B cells, the clearest EPR lines for oxidized Fe(3+)(Tp)_2_^+^ that were separated from other lines in the spectrum are the lines with *g* = 2.19 and *g* = 2.15 (Figure 2). A background line is superimposed on the high-field line for Fe(3+)(Tp)_2_^+^. Spectra for Fe(3+)(Tp)_2_^+^ in a solvent were used to calibrate the signal in the cells, as shown in Figure 2 (insert). The EPR spectrum for Fe(Tp)_2_^+^ indicates a low spin iron complex with rhombic g-values that are consistent with the structure for Fe(Tp)_2_^+^ (Figure 1). After comparing the peak height of the lines at 2.19 and 2.15 with these lines in the insert, it is estimated that the concentration of Fe(3+)(Tp)_2_^+^ was about 30 µM in 6 × 10^7^ BEAS-2B cells, where the spectrum for Fe(3+)(Tp)_2_^+^ added to BEAS-2B cells (25 scans) was corrected to compare to spectra with nine scans as in three of the four spectra in the insert. A concentration of 30 µM implies that much of Fe(3+)(Tp)_2_^+^ was oxidized in these cells. Iron was not removed from the Triapine complex. The extracellular volume was much larger than the intracellular volume. Therefore, the addition of 33.8 µM Fe(3+)(Tp)_2_^+^ to the culture medium and ~30 µM Fe(3+)(Tp)_2_^+^ inside the cells caused the cells to basically reach equilibrium across the membranes (i.e., the concentrations were approximately equal inside and outside the cells). If the cells had taken up all the Fe(3+)(Tp)_2_^+^, the intracellular concentration would have been very high because it all would have been concentrated in the much smaller intracellular volume. On average, Fe(3+)(Tp)_2_^+^ occurs in the ferric state. Therefore, the EPR method can be used to estimate the intracellular concentration of Fe(III)(Tp)_2_^+^. If Fe(3+)(Tp)_2_^+^ is not detected in other cells lines, the cells could be lysed to allow the reducing equivalents to dissipate. Other pharmacokinetic data imply that Triapine may be sequestered in cells/tissues given that 1.2% of the administered drug is recovered in urine [11,12]. A 33.8 µM concentration in the cells may not be unreasonable, particularly because the exposure time was much shorter compared with in vivo exposure times where blood levels are maintained over many days. Shorter exposure times require higher concentrations, whereas longer exposure times require lower levels. 

Also consistent with Fe(3+) being oxidized in cells under our conditions is that the iron sulfur clusters we studied in some cells are oxidized [13]. An additional signal at *g* = 4.3 attributed to non-heme iron was observed in BEAS-2B cells treated with Fe(3+)(Tp)_2_^+^ (not shown). This signal at *g* = 4.3 is not clearly resolved as expected for FeTf (Fe(3+)Tf), but some of this signal could be from Fe(3+)Tf where the superposition of lines from other non-heme iron signals obscures the expected resolved lines for Fe(3+)Tf. The detection of the low-spin EPR spectrum for Fe(3+)(Tp)_2_^+^ showed that the Fe(3+)(Tp)_2_^+^ complex is intact in BEAS-2B cells.

A second easily detectable signal is the line at *g* = 2.02 (actually the maximum of the S-shaped signal at *g* = 2.02), which is consistent with the signal for the [3Fe4S]^+1^ sites. The *g* = 2.02 signal is most often assigned to oxidized aconitase, but the S3 [3Fe4S] cluster from mitochondrial complex II could contribute as could the damaged [4Fe4S] centers [14]. At lower powers, a characteristic six-line spectrum from manganese was also apparent in the BEAS-2B cells [15]. The lines at *g* = 1.87 arise from the 4Fe4S cluster of the N3 center of complex I (*g* = 2.04, 1.93, 1.87) and from the mitochondrial electron-transferring flavoprotein (ETF) (*g* = 2.09, 1.87) [16,17,18]. The line at 1.87 is S-shaped, consistent with the shape for g-perpendicular for ETF. These signals provide evidence that Fe(3+)(Tp)_2_^+^ affects several sites in the mitochondria. The EPR spectrum for the BEAS-2B cells did not have any lines attributed to iron sulfur clusters except for a signal at *g* = 2.02 attributed to the 3Fe4S signal from aconitase [19].

### 2.2. Transfer of Fe from FeTf to Triapine

**Figure 3:**
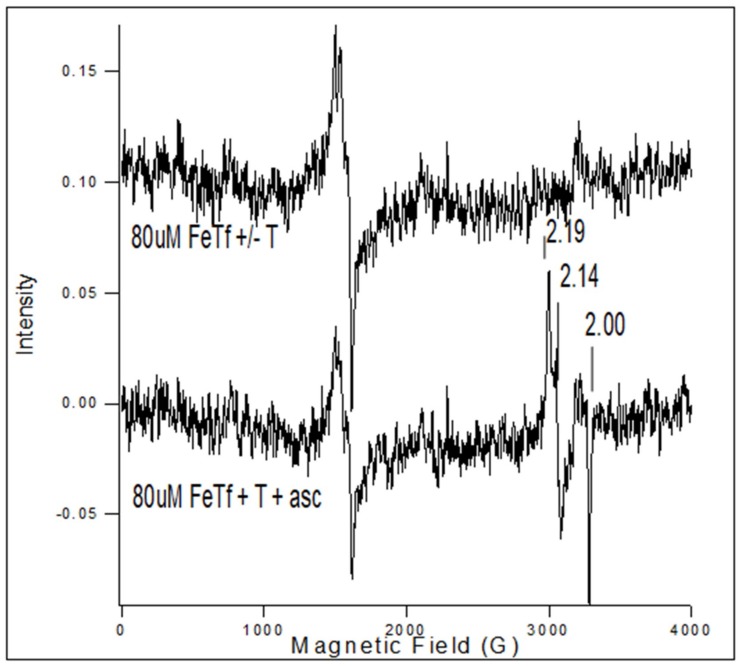
EPR spectrum for 80 µM FeTf with or without 30 µM Triapine (top spectrum) and FeTf plus Triapine plus ascorbate (bottom spectrum). The addition of ascorbic acid to FeTf in the presence of Triapine results in a decrease in the FeTf signal and the appearance of the FeT_2_^+^ signal.

The addition of ascorbic acid to FeTf in the presence of Tp resulted in a decrease in the FeTf signal and the appearance of the Fe(3+)(Tp)_2_^+^ signal (Figure 3). The iron signal from FeTf has a characteristic three-line spectrum at low field, as shown in Figure 3, while Fe(Tp)_2_^+^ has a low spin *S* = 1/2 spectrum with *g*-values at 2.19, 2.14, and 2.0, as marked in Figure 3.

Triapine did not remove iron from FeTf within 5 min (Figure 3, top spectrum), but upon addition of ascorbate (1 mg), Triapine removed iron from FeTf and formed Fe(3+)(Tp)_2_^+^ (Figure 3, bottom spectrum). This result suggests that after Fe in Fe-Tf is reduced from Fe(3+) to Fe(2+), the Fe(2+) is chelated by Triapine to form an Fe(2+)(Tp)**_2_** complex, which in the presence of oxygen is oxidized to Fe(3+)(Tp)_2_^+^. An EPR signal was detected for Fe(3+)(Tp)_2_^+^ (Figure 3, bottom spectrum). 

It is hypothesized that a similar reaction occurs in cells where Fe is sequestered as FeTf and endosomes are formed containing FeTf, transferrin receptor, and Triapine. The Fe in FeTf is reduced and released before binding to Triapine to form Fe(2+)(Tp)_2_, which is oxidized by oxygen or another oxidant to give Fe(3+)(Tp)_2_^+^, for which the EPR signal arises. If Fe(3+)-Triapine is formed in the endosomes, Fe-Triapine could be transported directly to the mitochondria, i.e., the “ kiss and run” hypothesis [20]. We acknowledge that this hypothesis is highly speculative, but if proven, the compartmentalization would enhance the formation of Fe(3+)(Tp)_2_^+^ in the mitochondria.

### 2.3. Additional Mechanism for Fe-Triapine: A Hypothesis

**Figure 4:**
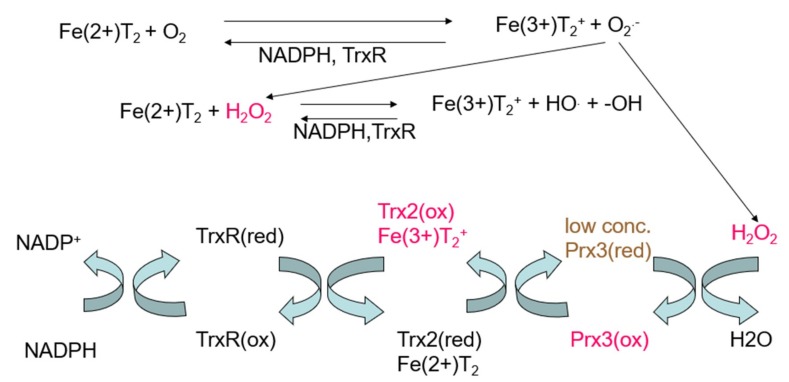
Reaction of Fe(2+)-Triapine, Fe(2+)(Tp)_2_, with oxygen to generate superoxide (O_2_^●−^), H_2_O_2_, and hydroxyl radical. Bottom: Coupled reactions for thioredoxin reductase (TrxR), thioredoxin (Trx), thioredoxin-2 (Trx2), and perioxiredoxin-3 (Prx3). Note: red = reduced, ox = oxidized.

To improve the efficacy of Triapine in future studies, two objectives are proposed. The first is to better define alternative or additional mechanisms for Triapine [4,21,22], for example, the generation of ROS in the mitochondria, which is supported by our previous studies [11,23,24]. The different mechanisms are monitored with different endpoints. The conventional mechanism involves inhibiting the conversion of ribonucleotides to deoxyribonucleotides and prevents replication of DNA for cell division [25,26]. The Gräslund model proposes a specific binding pocket for Triapine on the surface of ribonucleotide reductase, labilization of the diferric center in the R2 subunit of the protein by Triapine, and formation of Fe-Triapine and subsequently reactive oxygen species [26]. We have pieces of data that provide insight into the mechanism for mitochondrial damage. The EPR can be used to estimate the intracellular concentration of Fe(3+)-Triapine in cells, but it does not tell us about the distribution among subcellular compartments. So far, the only marker that we have to identify the redox effects in mitochondria is oxidation of peroxiredoxin-3 in the mitochondria, but not oxidation of peroxiredoxin-1 in the cytosol, after treatment with Triapine in A549 cells (Figure 4) [11,24]. Peroxiredoxin-3 (reduced) concentration was lowered from conversion of hydrogen peroxide (H_2_O_2_) to water, which drives peroxiredoxin-3 to the oxidized state (Figure 4). A549 cells were treated with 25 µM Triapine for 24 h [11]. A large amount of of H_2_O_2_ was generated, and peroxiredoxin-3(oxidized) (90%) was formed. Thioredoxin-2(oxidized) (40%) accumulated faster than it could be reduced, but thioredoxin reductase activity (thioredoxin reductase-1 plus thioredoxin reductase-2) was 100%.

A future study should compare the time course of the inhibition of ribonucleotide reductase with the oxidation of peroxiredoxin in the same cell line.

In summary, we hypothesize that an FeTp_2_^+^ complex is formed upon administration of Triapine. FeTp_2_^+^ accumulates in the mitochondria because the positive charge on the oxidized complex, Fe(3+)(Tp)_2_^+^, facilitates uptake. Under our study conditions, Fe(3+)(Tp)_2_^+^ was detected. This supports our hypothesis that Fe(3+)(Tp)_2_^+^ is taken up in the mitochondria because of the positive charge. We further hypothesize that oxidized Fe(3+)(Tp)_2_^+^ is reduced to Fe(2+)(Tp)_2_^+^ in the mitochondria by thioredoxin reductase, TrxR2 [23], and/or other mitochondrial reductants. The resulting oxidation of mitochondrial thioredoxin-2 and peroxiredoxin-3 [11,23] implies that the pro-oxidant effects of the redox cycling largely occur in the mitochondria; cytosolic thioredoxin-1 and peroxiredoxin-1 are not oxidized in Triapine-exposed cells, further indicating that the Triapine-induced oxidative stress is not widespread in cells but, rather, is confined largely to the mitochondria. Thioredoxin reductase-2 is an excellent reducing agent for Fe(3+)(Tp)_2_^+^ [23], and we detected Fe(3+)(Tp)_2_^+^ in BEAS-2B cells in this study. As such, it is feasible that rapid redox cycling is occurring in the mitochondria. When Fe(2+)(Tp)_2_ is oxidized it generates ROS (as shown in vitro [23] and depicted in Figure 4). The resulting generation of H_2_O_2_ drives the oxidation of peroxiredoxin-3 in human lung A549 cells, as depicted in Figure 4 [11]. Intertwined in this hypothesis is that control of iron uptake will influence the formation of Fe(3+)(Tp)_2_^+^ and the effectiveness of Triapine. It is speculated that mitochondrial uptake of a drug concentrates that drug in the mitochondria. As a result, there may be a change in mitochondrial potential, which should be investigated further. 

## 3. Materials and Methods 

### 3.1. Sample Preparation

Triapine was kindly provided by Vion Pharmaceuticals (New Haven, CT, USA), and stock solutions were prepared in 95% acetonitrile. Fe(3+)(Tp)_2_^+^ was prepared by mixing 2 volumes of 5 mM Triapine with 1 volume of freshly prepared aqueous 5 mM Fe(3+)Cl_3_ and gently rocking at room temperature for 1 h. For the EPR analysis of Fe(3+)(Tp)_2_^+^ (Figure 2, inset), a stock solution prepared in 87% dimethyl sulfoxide and 13% phosphate-buffered saline was further diluted to obtain the various Fe(3+)(Tp)_2_^+^ concentrations. 

BEAS-2B cells (human bronchial epithelial cells ATCC CRL-9609, Manassas, VA, USA) were grown at 37°C in humidified air containing 5% CO_2_ in Dulbecco’s Modified Eagle’s Medium with 25 mM HEPES (4-(2-hydroxyethyl)-1-piperazineethanesulfonic acid) and 4.5 g/L glucose (BioWhittaker 12-709F, Lonza Walkersville, Inc., Walkersville, MD, USA), supplemented with 10% LHC-9 medium (Invitrogen, Carlsbad, CA, USA), 10% fetal bovine serum (Valley Biomedical, Winchester, VA, USA), penicillin (100 U/mL), and streptomycin (100 µg/mL). For the cell experiments, Fe(3+)(Tp)_2_^+^ was generated as described above. Fe(3+)(Tp)_2_^+^ was added to the cell culture medium (to a final concentration of 33.8 µM) of BEAS-2B cells in six T75 flasks (grown to 50–70% confluence). After 24 h incubation, the medium was removed and discarded, and the cells were washed twice in cold Hank’s Balanced Salt Solution buffer (Invitrogen) to get rid of excess Fe(3+)(Tp)_2_^+1^. The cells were harvested, pooled, and suspended in 0.28 ml buffer. The cell suspension was placed in an EPR quartz tube (4 mm outside diameter) and frozen and stored in liquid nitrogen.

### 3.2. EPR Spectrometer

EPR spectra were obtained at liquid helium temperature using a Bruker E600 EleXsys spectrometer (Billerica, MA, USA) with an Oxford Instruments ESR-900 helium flow cryostat (Abingdon, UK) and either a Bruker DM0101 cavity or a Bruker ER4112SQG cavity. The samples were run at four microwave powers: 10, 16, 22, and 30 dB. The best results, based on a signal-to-noise ratio at 7 K, were obtained at 16 dB, where the 2Fe2S signal is slightly saturated. 

## Abbreviations

2Fe2S	Two-iron two-sulfur cluster
3Fe4S	Three-iron four-sulfur cluster
4Fe4S	Four-iron four-sulfur cluster
EPR	Electron paramagnetic resonance
ETF	Electron-transferring flavoprotein
Fe	Iron
Fe(3+)(Tp)_2_^+^	Fe(3+)-Triapine
FeTf	Iron transferrin
H_2_O_2_	Hydrogen peroxide
ROS	Reactive oxygen species
Tp	Triapine
Trx2	Mitochondrial thioredoxin reductase
